# Risk factors for COVID-19 in-hospital mortality in Argentina: A competing risk survival analysis

**DOI:** 10.1371/journal.pgph.0000816

**Published:** 2024-01-05

**Authors:** Sebastian Calonico, Juan Cruz Lopez Del Valle, Rafael Di Tella

**Affiliations:** 1 Department of Health Policy and Management, Mailman School of Public Health, ColumbiaUniversity, NYC, New York, United States of America; 2 Boston University, Boston, Massachusetts, United States of America; 3 Harvard Business School, Harvard University, Cambridge, Massachusetts, United States of America; 4 National Bureau of Economic Research, Cambridge, Massachusetts, United States of America; Fundacao Oswaldo Cruz, BRAZIL

## Abstract

COVID-19 poses dire threats for low and middle-income countries (LMICs). Yet, there remains limited rigorous evidence describing the characteristics and outcomes of hospitalized patients for LMICs, and often the evidence was based on small samples and/or unicentric. The objective of this study was to examine risk factors of COVID-19 mortality in Argentina, a hard-hit middle-income Latin American country. We analyze data on 5,146 COVID-19 patients from 11 centers across 10 cities in Argentina, making this one of the largest multi-centric retrospective observational descriptive studies in the LMICs. Information on demographics and co-morbidities was extracted from medical records. Outcomes of relevance consisted of whether the patient was discharged or deceased (as established in medical records), along with date of each event. We use survival models that account for competing risks. Median age was 60 years (IQR: 48–72), there were fewer women (40.8%) hospitalized than men (59.2%), and the most prevalent comorbidities were hypertension (40.9%), diabetes (20.0%) and obesity (19.1%). Patients were hospitalized for a median duration of 8 days (IQR: 5–13), and in-hospital mortality was 18.1%, though it varied substantially across health centers (95%CI: 17.1%-19.2%). Baseline characteristics most associated with in-hospital mortality were respiratory rate (adjusted HR = 3.6, 95%CI: 2.5–5.4 for ≥ 26 breathes/min), older age (adjusted HR = 2.5, 95%CI: 2.0–3.3 for the 80+ age group), and chronic kidney disease (adjusted HR = 2.2, 95%CI: 1.8–2.8). Associations were attenuated when survival models did not account for the competing risk of being discharged. We document lower mortality rates than those in prior studies, likely due to a lower prevalence of comorbidities amongst patients in our sample. Compared with standard Cox models, we find that, when using competing risk models, risk factors have a larger role in explaining COVID-19 mortality. Overall, we provide rigorous evidence describing the characteristics and outcomes of hospitalized patients for LMICs. Thus, our findings are useful to conduct a more accurate in-hospital monitoring of patient subgroups who may be at greater risk. They also provide valuable guidance for public health and policy efforts in Argentina and other developing countries.

## Introduction

Compared to high—income countries (HICs), COVID-19 poses dire threats for low and middle—income countries (LMICs) in part due to their weaker health care systems, higher poverty rates, urban overcrowding, large degree of informal labor [1], and poor state capacity [2–6]. First, inadequate health care systems, both in terms of quality and coverage, have made the pandemic difficult to handle [7–9]. While HICs have 4.68 hospital beds per 1000 people, LMICs only have around 1.28, suggesting that hospitalization from COVID-19 is especially detrimental for LMICs [10]. Second, overcrowding, unique dual-labor markets, and poverty have rendered physical distancing measures and policies ineffective in curbing COVID-19’s spread [11, 12]. These multifaceted susceptibilities compound, resulting in very high hospitalization and mortality rates [13, 14]. Despite calls for further research in LMICs [15], our knowledge on COVID-19 hospitalized patients is limited compared to HICs.

**Fig 1:**
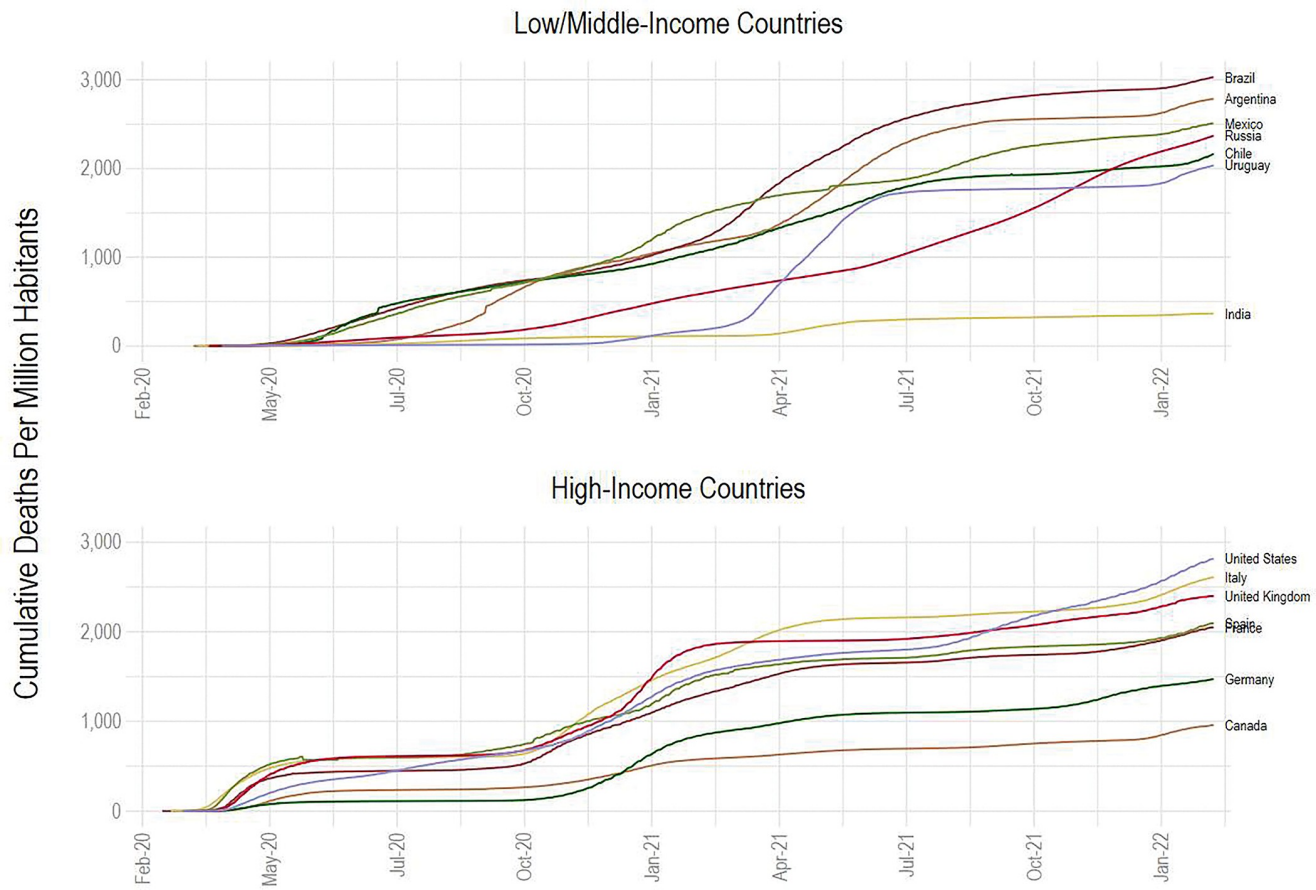
Cumulative COVID-19 deaths per million habitants for selected high- and middle-income countries. **Source:** Adapted using published data from Our World in Data [19].

Latin America is arguably the worst hit region by the COVID-19 pandemic in the world [16–18]. Argentina is among the top thirty countries with the highest cumulative deaths per million inhabitants [19] worldwide and has the fourth-highest mortality rate among all Latin American countries, closely behind Brazil. The mortality rate in Argentina is also greater than that of many HICs. As of February 2022, Argentina had 2,600 deaths per million habitants, higher than the US, UK, Spain, France, and Germany (Fig 1). COVID-19 has severely disrupted Argentina’s urban labor markets [20], especially in low-income neighborhoods where people already faced increased pre-existing conditions and other comorbidities [21], and lockdowns accelerated already high poverty rates [22]. Furthermore, the strain on the health care system has significantly affected the treatment and care of non-COVID-19 illnesses [23].

In spite of the social and economic consequences of COVID-19 in Argentina, rigorous evidence on risk factors, hospitalization, and mortality remains scarce. Previous work based on HICs [14] consistently found that COVID-19-related deaths were associated with age, gender and racial characteristics; as well as various medical conditions including diabetes and severe asthma. Evidence on role of these risk factors for LICs is still limited. Prior studies [24–26] in Argentina and other Latin American countries have mostly focused on the clinical features of COVID-19 based on a small number of patients [27–29], of unicentric nature [30], or mostly based on descriptive statistical analyses [31]. In the present study, we analyze data on COVID-19 patients from 11 centers spread across 10 cities in Argentina, which constitutes one of the largest multi-centric retrospective observational descriptive studies in low- and middle-income countries (S1 Table). Our statistical analysis addresses several important and timely gaps in our knowledge of COVID-19 hospitalization and mortality in Argentina, moving beyond standard time-to-event Cox models which were shown to result in biased associations, and potentially misleading policy recommendations [32].

### Background on the Epidemic in Argentina

The first detected COVID-19 case in Argentina occurred on March 3, 2020. Two weeks later, a decree by the national government established a mandatory preventive isolation for all residents of the country. The decree marked the beginning of a series of non-pharmaceutical preventative measures aimed to decrease the spread of virus. The lockdown eventually included closed schools, borders, and social-distance restrictions across many activities. Police controls were established in streets and highways to ensure the compliance of the lockdown measures, and this was enforced for several months. With the progression of the pandemic, some of these prevention strategies were updated by the health authorities.

Covid-19 infections in Argentina remained low and relatively constant until the incoming winter in mid-May 2020, when they started to increase rapidly despite strict ongoing lockdown measures. Around that time, the Ministry of Health established a reinforcement of the health system, increasing the number of ICU beds, professionally trained staff and critical care support infrastructure. New modular hospitals were opened in the areas where most severely affected by COVID-19. The country adapted its healthcare system by increasing critical care services, the deferral of nonurgent hospitalizations, the procurement of supplies, personal protective equipment, and the reduction of the use of hospital beds of high complexity.

After a first peak at the end of October 2020, the number of infections fell rapidly despite aperture of international borders. While vaccines became available in late January 2021, their distribution was limited to essential medical personnel until at least April that year, when they became available to those above 70 years old. A second peak of infections started at the end of March 2021, leading to a record number of deaths by mid-July. The major peak of infections happened in January 2021 with the Omnicron variant, this time without a similar peak in morbidity.

As of June 30, 2023, more than 10 million people tested positive for Covid-19, and 130,000 died. Deaths per 1 million population were 2,837, among the highest in the region, behind Peru (6,552), Chile (3,350) and Brazil (3,270).

## Methods

### Settings and study population

We analyzed in-hospital mortality due to COVID-19 as primary outcome using competing risk models, treating death due to COVID-19 and recovery as mutually exclusive competing events. A competing risk is an event whose occurrence precludes the occurrence of the event of interest. We included all PCR positive or clinically suspected COVID-19 patients admitted to 11 health centers located in 10 cities across Argentina. We distinguish between “PCR Positive” and “clinically suspected” COVID-19 patients since we do not have testing information in our dataset. Still, based on our conversations with the centers, we expect that all patients in our sample had a positive PCR test at the time of admission, as this was part of the strictly enforced official set of preventive measures imposed by the Argentina Health Ministry at the time of our study. The multicentric nature of our study is a key feature to study COVID-19 outcomes, as we are able to capture considerable heterogeneity between centers and regions, in part related to large inequities in the state of the healthcare system in Argentina. For instance, S1 Fig presents data on geographic variation of COVID-19 incidence at the province level.

The data, which tracks patients from time of hospitalization until being discharged or deceased, was extracted retrospectively from electronic medical records for all COVID-19 patients admitted from March 2020 through August 2021. In this period, the original strain (SARS-CoV-2) of Covid-19 was still predominant in Argentina. Data included information on demographics, comorbidities, oximetry, treatments received and outcomes (e.g., death vs. discharge). Data on symptoms and laboratory findings were only available for a subset of patients, and thus we do not use them in our analysis. Our main analytical sample included a total of 5,146 patients. Institutional Review Board at each health center approved the study. The present analysis was approved by the IRB at Columbia University and Harvard University (IRB-AAAT5728 and IRB20-189, respectively).

### Patient characteristics and outcomes of interest

Demographic factors included age (in years) and sex. Following the Centers for Disease Control and Prevention guidelines [31, 33] we extracted information on the following comorbidities: hypertension, smoking, diabetes, obesity, pulmonary diseases, other cardiovascular diseases, cancer and chronic kidney disease. Oximetry information included oxygen saturation (in percentage terms) and respiratory rate (in breaths per minute) at admission. Outcomes of relevance consisted of whether the patient was discharged or deceased (as established in medical records), along with date of each event.

### Statistical analysis

We first presented patient characteristics at baseline, overall and according to their outcome status (discharged vs. deceased). We initially extracted data on a total of 6,262 COVID-19 patients. Patients with incomplete records in any of the covariates of interest were excluded (n = 1,035). We also excluded patients (n = 81) whose hospital length of stay was missing, zero or larger than 56 days (4 times the 14 days disease cycle [34]) for a final analytical sample of 5,146 patients.

Studies investigating in-hospital mortality due to COVID-19 as primary outcome are usually based on the standard Cox proportional hazards model and the Kaplan–Meier estimator. These methods treat recovered patients as right-censored, implying that patients who recovered have similar risk of death compared to those still at risk (i.e., still hospitalized), an assumption known as noninformative censoring. While commonly employed, there are reasons to believe that recovered patients are not representative of those who are still admitted to the hospital in terms of COVID-19 mortality risk [32]. Thus, censoring recovered patients would induce bias and overestimation of survival curves, i.e. Kaplan Meier will estimate incidence of death with upwards biases [35, 36].

In this study, we treated death due to COVID-19 and recovery as mutually exclusive competing events. Therefore, we considered recovery as a competing risk for death rather than right-censoring. A competing risk is an event whose occurrence precludes the occurrence of the event of interest. Patients contributed observed time at risk beginning at their time of hospital admission. Time since hospital admission (in days) was considered as the time scale. We then used a sub-proportional hazard model of competing risks to study how baseline characteristics are associated with incident mortality among COVID-19 patients. The assumption of proportionality can be checked by testing statistical significance of interaction terms involving failure time and by computing Schoenfeld [37, 38]. In competing-risks regression, the goal is to estimate a cumulative incidence function indicating the probability of the event of interest happening before a given time. Competing-risks regression is semiparametric as the baseline subhazard is left unspecified, while the effects of covariates are assumed to be proportional [39–42]. We used Stata’s *stcrreg* function, which implements competing-risks regression based on Fine and Gray’s proportional subhazards model [43]. Our results are presented in terms of hazard ratios, with 95% confidence intervals calculated from standard errors clustered at the center level. We also presented cumulative incidence functions for mortality according to baseline characteristics.

### Role of the funding source

There is no funding affiliated with this work. The corresponding author had full access to all the data in the study and had final responsibility for the decision to submit for publication.

## Results

Patients’ baseline characteristics are summarized in Table 1, overall and across outcome categories (discharged or deceased). Of the total COVID-19 patients admitted, 4,213 (81.9%) were discharged, and 933 (18.1%) were deceased. The three largest centers contributed to almost half of our patient sample (center J with 1,116 (21.7%) patients, center G with 695 (13.5%) patients and center F with 533 (10.4%) patients). More than half of the admitted COVID-19 patients were men (59.2%), and median age was 60 years old (interquartile range 48–72). The three most prevalent comorbidities were hypertension (40.9%, 2,105 patients), diabetes (20%, 1,027 patients) and obesity (19.1%, 985 patients). Median oxygen saturation at admission was 95% (SD = 5.6, IQR = 92–97), and median respiratory rate measured at admission was 20 breaths per minute (SD = 5.6 and IQR = 18–22). Median length of hospital stay was 8 days (SD = 7.7 and IQR = 5–13). The distribution of those baseline characteristics varied across groups determined by the outcome (death and discharge status). In Table 2 we show that, compared to those who were discharged, deceased patients were more likely to be male, of older age, and more likely to have chronic conditions such as hypertension, obesity and chronic kidney disease. Furthermore, they were more likely to have had, at time of admission, low oxygen saturation of < 80 and high respiratory rate of ≥ 30, compared to patients who were discharged.

**Table 1:** Distribution of baseline characteristics overall and across outcome categories.

	Discharged		Dead		Total	
	N (%)		N (%)		N (100%)	
**Length of hospital stay**	8	5–12	6	11–18	8	5–13
**Gender**						
Female	1,756	41.7%	345	37.0%	2,101	40.8%
Male	2,457	58.3%	588	63.0%	3,045	59.2%
**Age, in years**						
< 50	1,397	33.2%	86	9.2%	1,483	28.8%
50–59	951	22.6%	109	11.7%	1,060	20.6%
60–69	871	20.7%	227	24.3%	1,098	21.3%
70–79	585	13.9%	250	26.8%	835	16.2%
> = 80	409	9.7%	261	28.0%	670	13.0%
**Comorbidities**						
Hypertension	1,605	38.1%	500	53.6%	2,105	40.9%
Smoking	210	5.0%	50	5.4%	260	5.1%
Obesity	796	18.9%	189	20.3%	985	19.1%
Diabetes	780	18.5%	247	26.5%	1,027	20.0%
Pulmonary Diseases	441	10.5%	130	13.9%	571	11.1%
Other Cardiovascular Diseases	463	11.0%	182	19.5%	645	12.5%
Cancer	197	4.7%	80	8.6%	277	5.4%
Chronic Kidney Disease	104	2.5%	78	8.4%	182	3.5%
**Oxygen Saturation, in percentage terms**						
< 80	48	1.1%	69	7.4%	117	2.3%
80–91	770	18.3%	275	29.5%	1,045	20.3%
> = 92	3,395	80.6%	589	63.1%	3,984	77.4%
**Respiratory Rate, in breaths per minute**						
< 20	1,905	45.2%	214	22.9%	2,119	41.2%
20–29	2,161	51.3%	609	65.3%	2,770	53.8%
> = 30	147	3.5%	110	11.8%	257	5.0%
**Center**						
Center A	376	8.9%	61	6.5%	437	8.5%
Center B	100	2.4%	86	9.2%	186	3.6%
Center C	94	2.2%	20	2.1%	114	2.2%
Center D	330	7.8%	83	8.9%	413	8.0%
Center E	250	5.9%	109	11.7%	359	7.0%
Center F	434	10.3%	99	10.6%	533	10.4%
Center G	602	14.3%	93	10.0%	695	13.5%
Center H	209	5.0%	29	3.1%	238	4.6%
Center I	153	3.6%	25	2.7%	178	3.5%
Center J	1,064	25.3%	52	5.6%	1,116	21.7%
Center K	601	14.3%	276	29.6%	877	17.0%
**Total**	4,213	81.9%	933	18.1%	5,146	100%

**Note:** All data are presented as n (%) except for length of hospital stay presented as median (IQR).

**Table 2:** Distribution of baseline characteristics between outcome categories, and their differences.

	(1)	(2)	(3)
Variable	Discharged	Dead	Difference
Length of hospital stay	9.682	13.555	3.873***
	(6.836)	(9.926)	(0.000)
Sex (Male)	0.583	0.630	0.047***
	(0.493)	(0.483)	(0.007)
Age	56.705	70.130	13.424***
	(17.336)	(14.327)	(0.000)
Hypertension	0.381	0.536	0.155***
	(0.486)	(0.499)	(0.000)
Other Cardiovascular Diseases	0.110	0.195	0.085***
	(0.313)	(0.396)	(0.000)
Diabetes	0.185	0.265	0.080***
	(0.388)	(0.441)	(0.000)
Pulmonary Diseases	0.105	0.139	0.035***
	(0.306)	(0.346)	(0.005)
Cancer	0.047	0.086	0.039***
	(0.211)	(0.280)	(0.000)
Smoking	0.050	0.054	0.004
	(0.218)	(0.225)	(0.644)
Obesity	0.189	0.203	0.014
	(0.392)	(0.402)	(0.346)
Chronic Kidney Disease	0.025	0.084	0.059***
	(0.155)	(0.277)	(0.000)
Oxygen Saturation	94.028	90.966	-3.062***
	(4.733)	(8.034)	(0.000)
Respiratory Rate	20.287	22.865	2.578***
	(5.627)	(5.277)	(0.000)
Observations	4,213	933	5,146

Notes: standard errors in parentheses.

*** statistically significant at 1% level.

**Fig 2:**
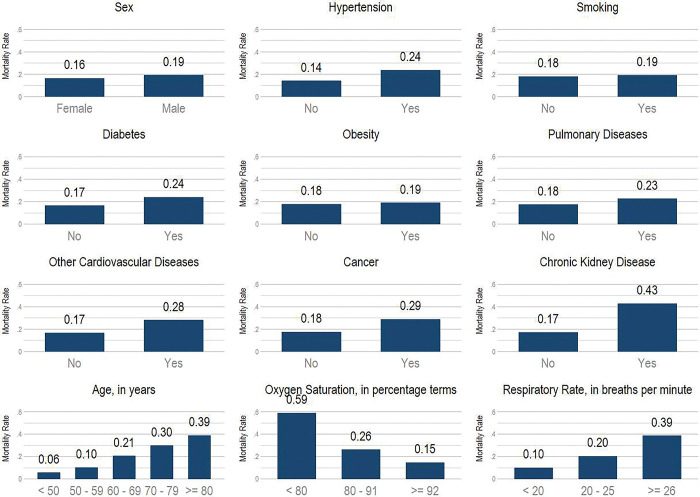
COVID-19 mortality rates across categories of baseline characteristics. **Source:** Own calculations.

**Fig 3:**
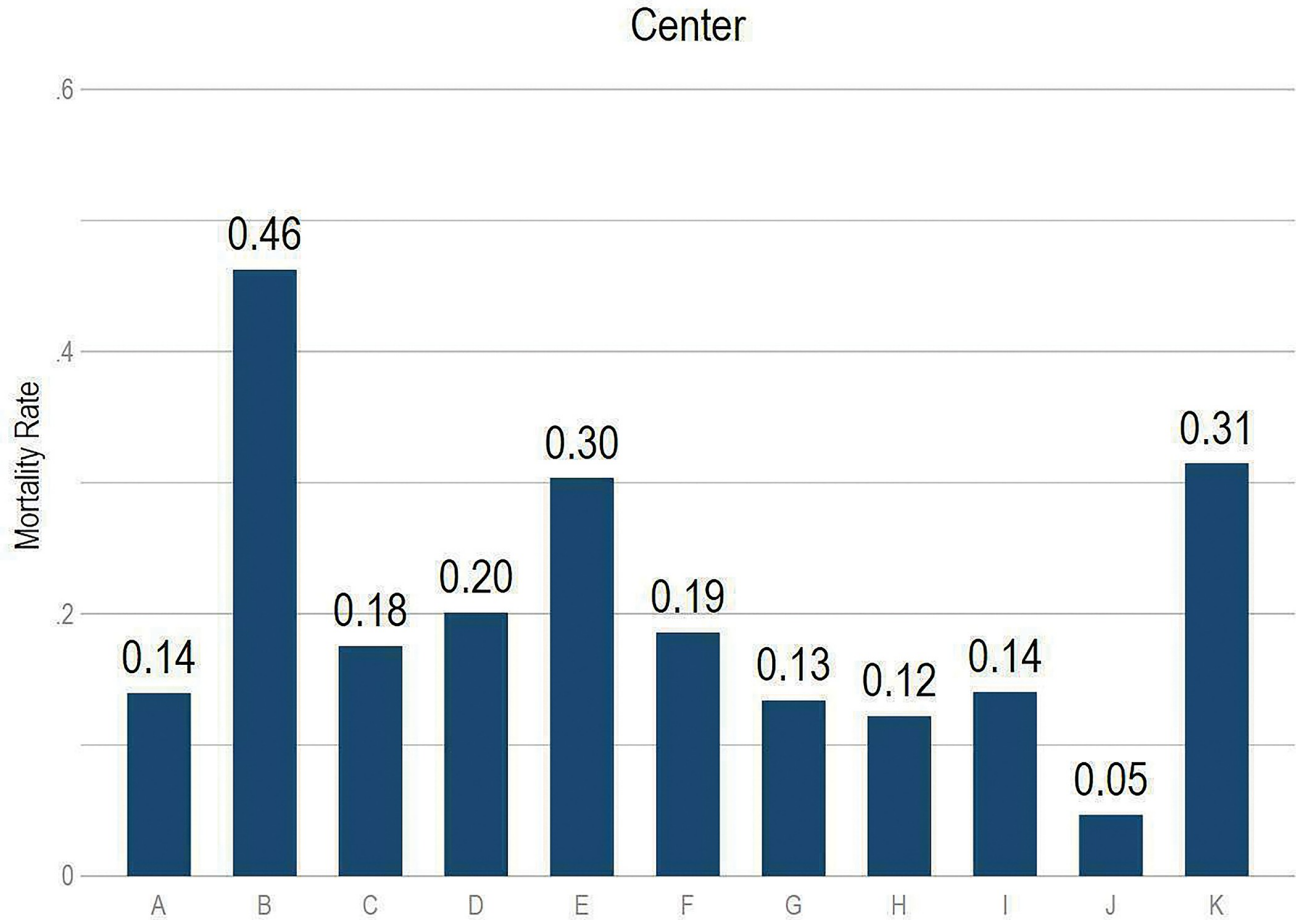
COVID-19 mortality rates across centers. **Source:** Own calculations.

Mortality rates differed across categories of patients’ characteristics (Fig 2). For example, mortality rate for men was 3 percentage points (p.p.) higher than for women (19.3% vs 16.4%, respectively). Compared to the mortality rate among those ages < 50, the mortality rates were only 4.5 p.p. higher among those ages 50–59, but were 24.8 and 33.2 p.p. higher among those ages 70–79 years old and 80+ years old respectively. Furthermore, mortality rates varied across comorbidity status. For example, mortality rates were more than 25 p.p. higher among those with chronic kidney disease (vs. not), 11 p.p. higher among those with cancer (vs. not), and 9.5 p.p. higher among those with hypertension (vs. not). We also observed substantial heterogeneity in mortality rates across centers (Fig 3).

**Fig 4:**
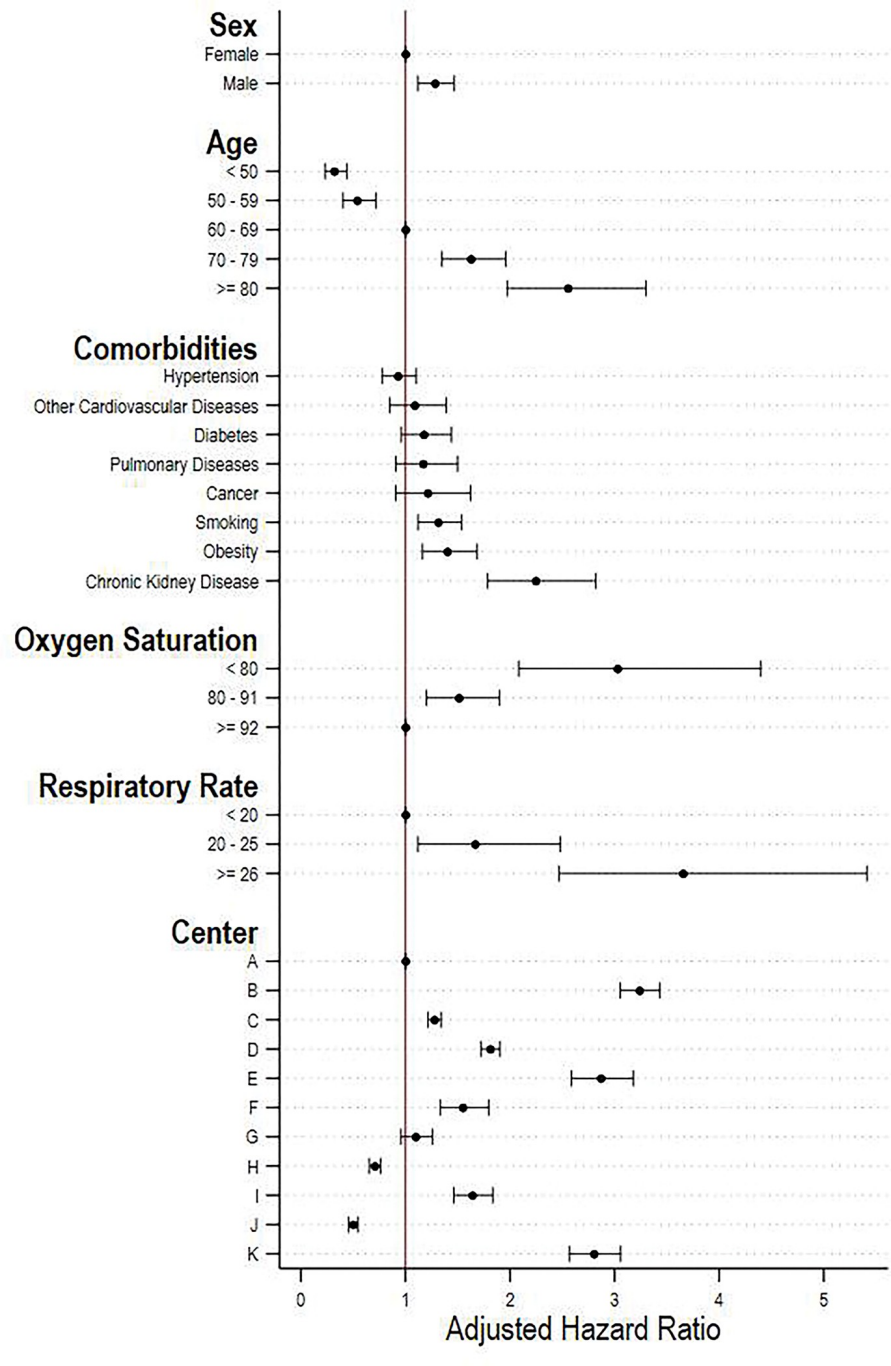
Associations between baseline characteristics and mortality. **Note:** Competing risk regression models. 95% confidence intervals calculated from standard errors clustered at the center level.

Next, we discuss the main findings from our competing risk models for the associations between baseline patient characteristics and time to death, while treating discharge as a competing event. **Fig 4** is a forest plot displaying the coefficients as hazard ratios. Several baseline characteristics were significantly associated with the hazard of mortality. In particular, the three characteristics that showed the highest effect on mortality were older age, having chronic kidney disease and respiratory rate > 25. Being 80+ (vs. 60–69 years of age) was associated with 150% higher probability of death (adjusted HR 2.5, 1.9–3.3), having chronic kidney disease (vs. not) was associated with 120% higher probability of death (adjusted HR 2.2, 95% CI 1.8–2.7), and having a respiratory rate ≥ 26 (vs. < 20) was associated with 260% higher probability of death (adjusted HR 3.6, 95% CI 2.46–5.4). Furthermore, being male and having lower oxygen saturation was associated with higher hazard of mortality. We also present all the estimation results in **Table 3**.

**Table 3:** Adjusted hazard ratios from competing risk model of mortality.

	(1)	(2)	(3)
VARIABLES	Odds Ratio	SE	95% CI
Male	1.282***	(0.089)	1.119–1.467
Age (years), < 50	0.319***	(0.052)	0.231–0.439
Age (years), 50–59	0.538***	(0.079)	0.403–0.717
Age (years), 70–79	1.627***	(0.155)	1.349–1.961
Age (years), > = 80	2.553***	(0.334)	1.976–3.300
Hypertension	0.928	(0.082)	0.780–1.103
Other Cardiovascular Diseases	1.088	(0.136)	0.852–1.390
Diabetes	1.176	(0.122)	0.960–1.440
Pulmonary Diseases	1.168	(0.149)	0.910–1.499
Cancer	1.213	(0.180)	0.907–1.622
Smoking	1.313***	(0.106)	1.121–1.539
Obesity	1.400***	(0.133)	1.162–1.685
Chronic Kidney Disease	2.245***	(0.262)	1.787–2.821
Oxygen Saturation (%), < 80	3.028***	(0.577)	2.084–4.399
Oxygen Saturation, (%), 80–91	1.511***	(0.176)	1.202–1.899
Respiratory Rate (breaths per minute), 20–25	1.666**	(0.338)	1.119–2.480
Respiratory Rate (breaths per minute), > = 26	3.654***	(0.733)	2.467–5.413
Center B	3.237***	(0.097)	3.053–3.432
Center C	1.276***	(0.032)	1.214–1.341
Center D	1.811***	(0.047)	1.722–1.905
Center E	2.869***	(0.150)	2.588–3.179
Center F	1.548***	(0.117)	1.335–1.795
Center G	1.098	(0.077)	0.957–1.260
Center H	0.707***	(0.028)	0.654–0.765
Center I	1.639***	(0.095)	1.463–1.836
Center J	0.499***	(0.024)	0.455–0.548
Center K	2.802***	(0.125)	2.567–3.058
Observations	5,146		

Robust Standard Errors in parentheses

*** p<0.01

** p<0.05

* p<0.1

**Fig 5:**
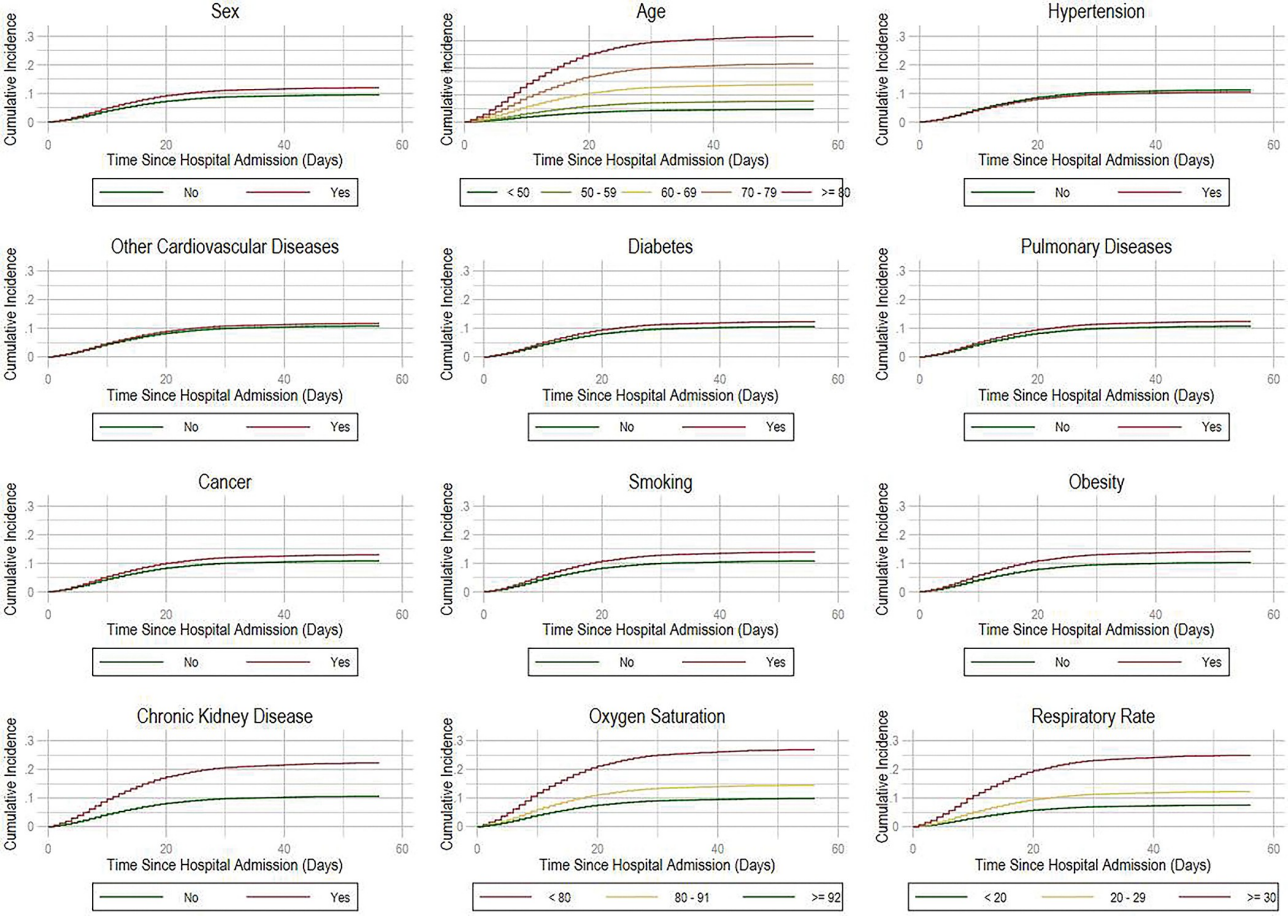
Cumulative incidence functions for deceased patients. **Source:** Own calculations.

We next present evidence supporting the proportional sub-hazard assumption used to construct our competing risk model. Proportional sub-hazards imply that the relative sub-hazard (that is, the coefficients) are fixed over time, and this assumption would be violated if a time interaction proved significant. We provide graphical evidence of this assumption in **Fig 5**, where we display cumulative incidence functions for mortality according, showing no time interaction according to baseline characteristics.

**Fig 6:**
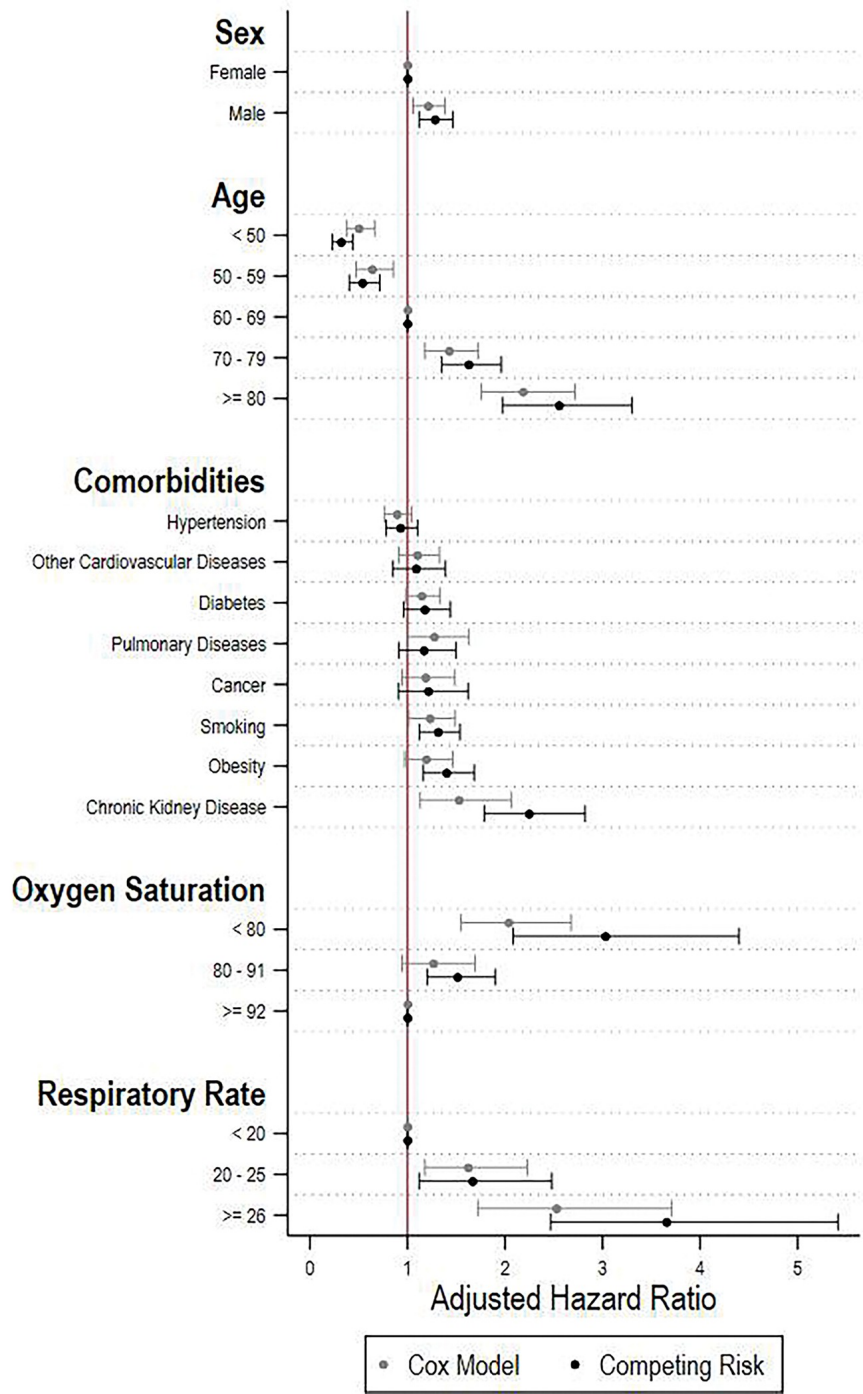
Competing risk analysis vs Cox model. **Notes:** 95% confidence intervals calculated from standard errors clustered at the center level. Centers not showed for the sake of clarity. **Source:** Own calculations.

Finally, we illustrate the performance of our competing risk model. In **Fig 6** we present a forest plot with the coefficients from a conventional Cox proportional Hazards regression (i.e. that do not treat hospital discharge as a competing event) together with our original estimates. Overall, associations between baseline patient characteristics and death hazard were attenuated when using a Cox Model, with age, chronic kidney disease, and respiratory rate showing the largest differences.

## Discussion

The present study is one of the largest multicentric retrospective observational descriptive cohort studies on COVID-19 patients in low- and middle-income countries, and in Latin America in particular. We find an overall mortality rate of 18.1%, with age, respiratory rate, and chronic kidney disease significantly associated with higher rate of mortality. An overall in-hospital mortality rate of 18.1% is lower than those reported in previous studies carried both in HICs and LMICs, ranging from 24.5% [26] to 30.1% [30]. Still, 18.1% mortality rate is above those found by other studies on hospitalized patients carried in Argentina (9.8% [29], 11.0% [27] and 12.4% [31]) reported in **S1 Table**. These findings highlight the relevance of analysis based on multi-center data. We observed substantial heterogeneity in mortality rates across healthcare centers (with the lowest mortality rate at 4.7% and the highest at 46.2%). Additionally, such differences are in line with prior literature suggesting that Argentina and other South American countries fare better in terms of comorbidities and cardiovascular health compared with their counterparts from the US and other high-income countries [44]. Furthermore, the prevalence of comorbidities in our sample might as well reflect the fact that the population in Argentina is younger than the average HIC, and that Argentina’s per capita GDP is higher than the average LMIC [45, 46]. Further research is needed to uncover the reasons behind this difference. Even though we do not have information about Covid-19 vaccination status in our data, we do not see this as playing a major role, since vaccines were not widely available in Argentina until April of 2021. Given the combination of the length of our study period (date of admission) with the age composition of the patients, the majority of them were not eligible for vaccination at the time of their hospital admission.

Our findings regarding the role of patient characteristics on COVID-19 mortality were mostly in line with prior studies. This was particularly true for age [47], respiratory rate (which is a good indicator of the state of the patient at the time of hospitalization) [48], and oxygen saturation [49]. Among all comorbidities, chronic kidney disease had the largest negative effect on mortality, likely by rendering the immunosuppressive system extremely fragile [50]. Other comorbidities, such as hypertension, diabetes, pulmonary diseases, other cardiovascular diseases and cancer, were not associated with mortality in models adjusted for potential confounders. The lack of association between pulmonary disease and mortality while surprising, is rather increasingly confirmed by epidemiological data [51, 52].

More importantly, by treating discharge as a competing event, our findings suggest stronger associations (larger hazard ratios) between risk factors and mortality compared to when discharge is treated as being censored (as in standard Cox regression). In other word, the strength of risk factors is attenuated when we did not use a competing risk model. While a strict comparison with prior studies is not possible given differences in the baseline characteristics considered, how continuous variables are discretized, and the number of patients included, this evidence highlights the needs of carefully conducted studies dealing with potential biases in order to properly inform policy.

Our study has some limitations that are worth noting. Being on a ventilator or admitted to the intensive care unit are likely important predictors of mortality, but they were not included in the hospital records in a standardized way across center, and thus could not be extracted. Detailed laboratory findings (including PCR tests results) that could have been used by the physicians at the time of deciding patients’ treatment were not always available, and thus were not analyzed. We also lack socioeconomic data, which could have played a role explaining COVID-19 deaths. Our study also has several important strengths, including its large sample size compared to studies from other middle-income countries in Latin America. The multicentric nature of our study not only reveals a considerable heterogeneity in COVID-19 outcomes between centers, but this variation captures inequities in the state of the healthcare system in Argentina. Finally, on the methodological side, the use of competing risk regression stronger associations between patients’ risk factors and risk of mortality.

This study fills an important gap in our knowledge on the drivers of COVID-19 mortality among patients in Argentina. In light of the continuing threat of COVID-19, further studies employing rigorous analytical techniques are needed in Argentina and other hard-hit middle-income Latin American countries.

## Supporting information

S1 Table Comparison with prior studies. (DOCX)

S1 Fig COVID-19 incidence across Argentina. Variation at the province level.
(TIF)
