# Supplementary material for: Risk factors for COVID-19 in-hospital mortality in Argentina: A competing risk survival analysis
Source: PLOS Glob Public Health. 2024 Jan 5;4(1):e0000816. doi: 10.1371/journal.pgph.0000816 (PMC10769012; doi:10.1371/journal.pgph.0000816)
Supplement: S1 Table — (DOCX) [file pgph.0000816.s001.docx]

**S1 Table.** Comparison with prior studies

| **Country** | **Paper** | **Centers** | **Patients** | **Mortality** | **Diabetes** | **Obesity** | **HTA** | **CKD** | **Age** | **Methods** |
| --- | --- | --- | --- | --- | --- | --- | --- | --- | --- | --- |
| Germany | Günster et al. (2021) [^[24]^](https://journals.plos.org/plosone/article?id=10.1371/journal.pone.0255427) | - | 8,679 | 24.9% | 30.5% | - | 56.7% | - | 68* | LR |
| Netherlands | Nijman et al. (2021) [^[28]^](https://journals.plos.org/plosone/article?id=10.1371/journal.pone.0249231) | 5 | 1,006 | 24.6% | 22.8% | - | 38.9% | 11.2% | 69 | CR |
| UK + Italy | Hewitt et al. (2020) [^[25]^](https://www.thelancet.com/journals/lanpub/article/PIIS2468-2667(20)30146-8/fulltext) | 11 | 1,564 | 27.2% | 26.5% | - | 51.4% | - | - | CM |
| United States | Richardson et al. (2020) [^[26]^](https://jamanetwork.com/journals/jama/fullarticle/2765184) | 12 | 5,700 | 24.5% | 33.8% | 41.7% | 56.6% | 5.0% | 63 | NA |
| Argentina | Barra et al. (2021) [^[31]^](https://www.medrxiv.org/content/10.1101/2021.07.30.21261220v2.full.pdf) | 4 | 668 | 12.4% | 17.1% | 3.1% | 33.1% | 4.5% | - | NA |
| Argentina | Cordova et al. (2021) [^[27]^](https://journals.plos.org/plosone/article?id=10.1371/journal.pone.0258260) | 19 | 809 | 11.0% | 17.1% | 23.2% | 31.9% | 4.1% | 53 | LR |
| Argentina | [Yacobitti et al. (2021)](https://www.nature.com/articles/s41598-021-87552-w) [^[29]^](https://www.nature.com/articles/s41598-021-87552-w) | 14 | 1,495 | 9.8% | 15.0% | 7.0% | 21.0% | 3.0% | 48.9* | LR |
| Brazil | [Martins-Filho et al. (2021)](https://pubmed.ncbi.nlm.nih.gov/33215578/#:~:text=Moreover%2C%20infectious%20disease%20(OR%3A,%2D19%20in%2Dhospital%20death.) [^[28]^](https://www.scielo.br/j/eins/a/WKfHm3xHqFFxqTcxLVDSd7b/?format=html&lang=pt) | - | 1,207 | 29.2% | 24.5% | 9.0% | 36.1% | 1.8% | 60 | LR |
| Mexico | [Olivas-Martínez et al. (2021)](https://pubmed.ncbi.nlm.nih.gov/33534813/#:~:text=The%20main%20risk%20factors%20associated,%25CI%203.26%2D7.31).) [^[30]^](https://academic.oup.com/cid/article/73/1/1/5920244?login=true) | 1 | 800 | 30.1% | 26.0% | - | 30.0% | 3.0% | 51.9* | NA |
| **Average** | **-** | **9.4** | **2,436.4** | **21.5%** | **23.7%** | **16.8%** | **39.5%** | **4.7%** | **-** | **-** |
| **Average Argentina** | **-** | **12.3** | **991** | **11.1%** | **16.4%** | **11.1%** | **28.7%** | **3.9%** | **-** | **-** |
| **Average (- Argentina)** | - | **7.3** | **3,159** | **26.8%** | **27.4%** | **25.4%** | **45.0%** | **5.3%** | **-** | **-** |
| **Argentina** | **Our Study** | **11** | **5,146** | **18.1%** | **20.0%** | **19.1%** | **40.9%** | **3.5%** | **60** | **CR** |
| **Notes:** Grasselli et al. (2020) [^[s1]^](https://jamanetwork.com/journals/jama/article-abstract/2763188) due to statistics reported being for ICU patients. Angulo et al (2021) ^[[s2]](https://jamanetwork.com/journals/jamanetworkopen/article-abstract/2774584)^, Asch et al (2021) ^[[s3]](https://jamanetwork.com/journals/jamainternalmedicine/article-abstract/2774572)^ and Nguyen et al. (2021) [^[s4]^](https://jamanetwork.com/journals/jamanetworkopen/article-abstract/2777028) excluded due to being studies order of magnitudes larger (42,604; 38,517 and 192,505 patients respectively). Cummings et al. (2020) [^[s5]^](https://www.sciencedirect.com/science/article/pii/S0140673620311892?casa_token=sSj2Ny99_YgAAAAA:mke_plNra4PaDx06hr6Di3eZl8nN-sE5GHWjwxZOwB8DjVj0KGnZ9kdPtJmefTAcIh6ctrzEbEBO) excluded due to statistics reported being for a subsample of 257 critically ill patients. Garibaldi et al. (2020) [^[s6]^](https://www.ncbi.nlm.nih.gov/pmc/articles/PMC7530643/) excluded due to “our more complete observation of discharge outcome”. Teich et al. (2020) [^[s7]^](https://www.scielo.br/j/eins/a/WKfHm3xHqFFxqTcxLVDSd7b/?lang=en&format=html) excluded due to only 72 patients hospitalized. Chen et al (2021) [^[s8]^](https://www.sciencedirect.com/science/article/pii/S2095809921004239) excluded due to outcome reported being for deferred patients. Kirillov (2021) [^[s9]^](https://journals.sagepub.com/doi/full/10.1177/21501327211008050) excluded due to statistics reported being for deceased patients. Munblit et al. (2021) [^[s10]^](https://academic.oup.com/cid/article/73/1/1/5920244?login=true) excluded due to statistics being reported for PCR negative patients. * refers to mean age, otherwise is median. LR refers to Logistic Regression, CR refers to Competing Risk, CM refers to Cox Model, NA refers to No Analysis. | | | | | | | | | | |

**References**

1. Grasselli, G., Pesenti, A., & Cecconi, M. (2020). Critical care utilization for the COVID-19 outbreak in Lombardy, Italy: early experience and forecast during an emergency response. *Jama, 323(16)*, 1545-1546.
2. Angulo, F. J., Finelli, L., & Swerdlow, D. L. (2021). Estimation of US SARS-CoV-2 infections, symptomatic infections, hospitalizations, and deaths using seroprevalence surveys*. JAMA network open, 4(1)*, e2033706-e2033706.
3. Asch, D. A., Sheils, N. E., Islam, M. N., Chen, Y., Werner, R. M., Buresh, J., & Doshi, J. A. (2021). Variation in US hospital mortality rates for patients admitted with COVID-19 during the first 6 months of the pandemic. *JAMA internal medicine, 181(4)*, 471-478.
4. Nguyen, N. T., Chinn, J., Nahmias, J., Yuen, S., Kirby, K. A., Hohmann, S., & Amin, A. (2021). Outcomes and mortality among adults hospitalized with COVID-19 at US medical centers. *JAMA Network Open*, *4*(3), e210417-e210417.
5. Cummings, M. J., Baldwin, M. R., Abrams, D., Jacobson, S. D., Meyer, B. J., Balough, E. M., ... & O'Donnell, M. R. (2020). Epidemiology, clinical course, and outcomes of critically ill adults with COVID-19 in New York City: a prospective cohort study. *The Lancet*, *395*(10239), 1763-1770.
6. Garibaldi, B. T., Fiksel, J., Muschelli, J., Robinson, M. L., Rouhizadeh, M., Perin, J., ... & Gupta, A. (2021). Patient trajectories among persons hospitalized for COVID-19: a cohort study. *Annals of internal medicine*, *174*(1), 33-41.
7. Teich, V. D., Klajner, S., Almeida, F. A. S. D., Dantas, A. C. B., Laselva, C. R., Torritesi, M. G., ... & Cendoroglo, M. (2020). Epidemiologic and clinical features of patients with COVID-19 in Brazil. *Einstein (Sao Paulo)*, *18*.
8. Chen, S., Sun, H., Heng, M., Tong, X., Geldsetzer, P., Wang, Z., ... & Bärnighausen, T. (2021). Factors Predicting Progression to Severe COVID-19: A Competing Risk Survival Analysis of 1753 Patients in Community Isolation in Wuhan, China. *Engineering*.
9. Kirillov, Y., Timofeev, S., Avdalyan, A., Nikolenko, V. N., Gridin, L., & Sinelnikov, M. Y. (2021). Analysis of Risk Factors in COVID-19 Adult Mortality in Russia. *Journal of Primary Care & Community Health*, *12*, 21501327211008050.
10. Munblit, D., Nekliudov, N. A., Bugaeva, P., Blyuss, O., Kislova, M., Listovskaya, E., ... & Glybochko, P. (2021). Stop COVID cohort: an observational study of 3480 patients admitted to the Sechenov university hospital network in Moscow City for suspected coronavirus disease 2019 (COVID-19) infection. Clinical Infectious Diseases, 73(1), 1-11.
