# Supplementary figures and images for: Risk factors for COVID-19 in-hospital mortality in Argentina: A competing risk survival analysis
Source: PLOS Glob Public Health. 2024 Jan 5;4(1):e0000816. doi: 10.1371/journal.pgph.0000816 (PMC10769012; doi:10.1371/journal.pgph.0000816)

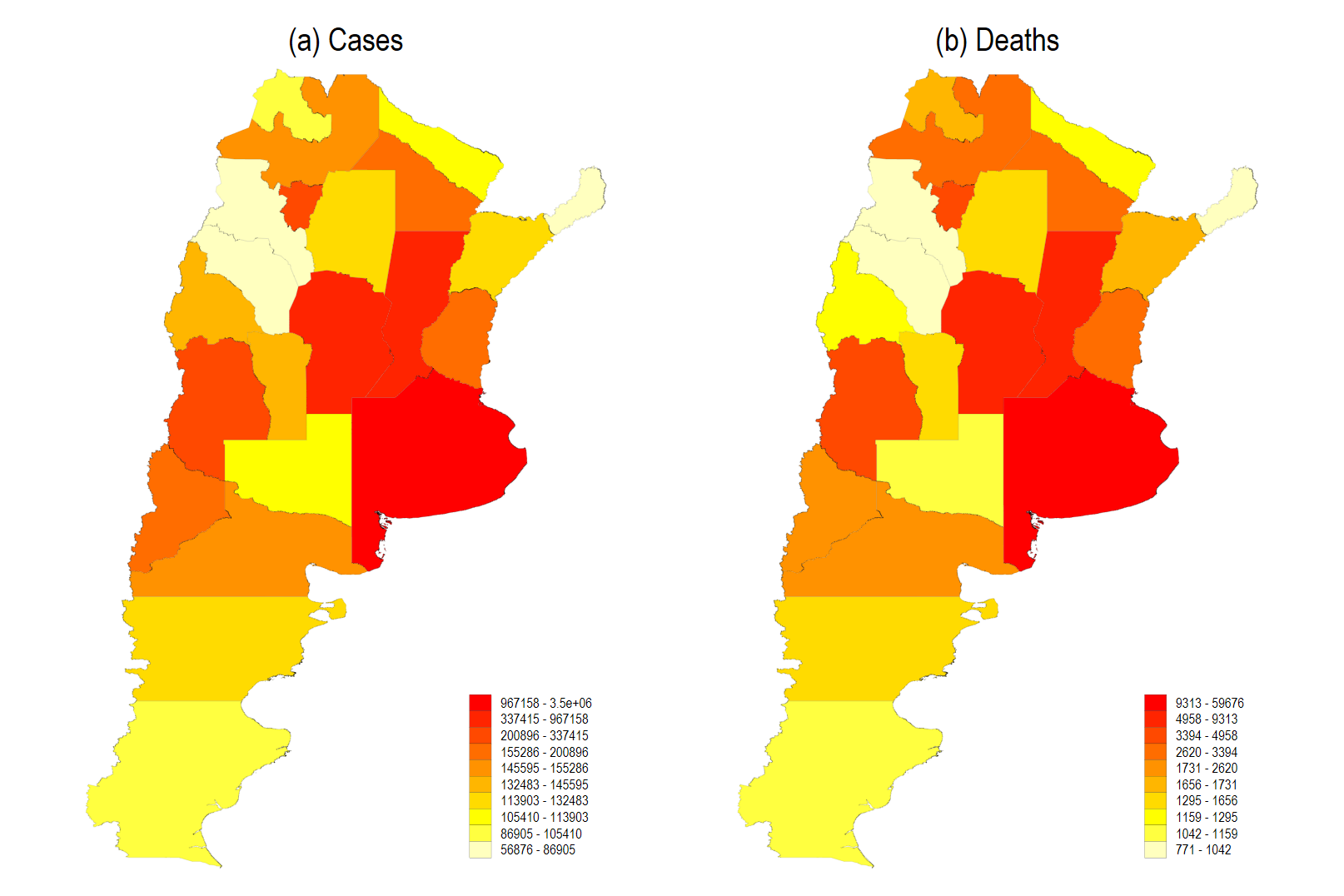

Supplement: S1 Fig — Variation at the province level. (TIF) [file pgph.0000816.s002.tif]
